# Alternating current stimulation promotes neurite outgrowth and plasticity in neurons through activation of the PI3K/AKT signaling pathway: Neurite growth enhanced by alternating current stimulation via PI3K/AKT pathway

**DOI:** 10.3724/abbs.2023238

**Published:** 2023-10-09

**Authors:** Hao Zhong, Cong Xing, Mi Zhou, Zeyu Jia, Song Liu, Shibo Zhu, Bo Li, Hongjiang Yang, Hongpeng Ma, Liyue Wang, Rusen Zhu, Zhigang Qu, Guangzhi Ning

**Affiliations:** 1 International Science and Technology Cooperation Base of Spinal Cord Injury Tianjin Key Laboratory of Spine and Spinal Cord Injury Department of Orthopedics Tianjin Medical University General Hospital Tianjin 300052 China; 2 Department of Spine Surgery Tianjin Union Medical Center Tianjin 300121 China; 3 College of Electronic Information and Automation Advanced Structural Integrity International Joint Research Center Tianjin University of Science and Technology Tianjin 300222 China

**Keywords:** electrical stimulation, cortical neurons, neural regeneration, neurite outgrowth and plasticity, alternating current stimulation.

## Abstract

As a commonly used physical intervention, electrical stimulation (ES) has been demonstrated to be effective in the treatment of central nervous system disorders. Currently, researchers are studying the effects of electrical stimulation on individual neurons and neural networks, which are dependent on factors such as stimulation intensity, duration, location, and neuronal properties. However, the exact mechanism of action of electrical stimulation remains unclear. In some cases, repeated or prolonged electrical stimulation can lead to changes in the morphology or function of the neuron. In this study, immunofluorescence staining and Sholl analysis are used to assess changes in the neurite number and axon length to determine the optimal pattern and stimulation parameters of ES for neurons. Neuronal death and plasticity are detected by TUNEL staining and microelectrode array assays, respectively. mRNA sequencing and bioinformatics analysis are applied to predict the key targets of the action of ES on neurons, and the identified targets are validated by western blot analysis and qRT-PCR. The effects of alternating current stimulation (ACS) on neurons are more significant than those of direct current stimulation (DCS), and the optimal parameters are 3 μA and 20 min. ACS stimulation significantly increases the number of neurites, the length of axons and the spontaneous electrical activity of neurons, significantly elevates the expression of growth-associated protein-43 (GAP-43) without significant changes in the expression of neurotrophic factors. Furthermore, application of PI3K/AKT-specific inhibitors significantly abolishes the beneficial effects of ACS on neurons, confirming that the PI3K/AKT pathway is an important potential signaling pathway in the action of ACS.

## Introduction

The economic impact of central nervous system-related diseases on patients is substantial because permanent disability leads to a high estimated lifetime cost of treatment per patient [ 1, 2] . Although various interventions have been developed, most strategies are currently palliative because the intrinsic regenerative capacity is limited. Therefore, it is necessary to explore the mechanisms that activate neurons and verify that the underlying mechanisms have the potential to improve and broaden the therapeutic applications of treatments for central nervous disorders.

The application of electrical stimulation (ES) to the nervous system has been examined for hundreds of years [3]. The development of related disciplines has expanded the methods for interfacing. ES has been widely applied to modulate the central nervous system, such as through deep brain stimulation (DBS) [4] and spinal cord stimulation (SCS) [5]. As an invasive neurosurgical procedure, DBS delivers electrical pulses to a specific area through implanted electrodes [6]. DBS provides a novel method to facilitate brain function across the brain by modulating aberrant or desynchronized activity or activating dormant networks. The effectiveness of DBS in chronic emotional regulation and behavioral control in individuals who have incurred severe traumatic brain injury has been demonstrated in a clinical study [7]. Spinal cord injury (SCI) leads to catastrophic neurological impairment and thereby causes deficits in sensory/motor capacity and permanent paralysis [8]. SCS activates specific pathways and segmentally modulates neuroglial interactions. Because the amplitudes of the applied current are considerably below the sensory perception threshold, SCS is a paresthesia-free therapy. SCS has been widely used in the treatment of chronic pain and functional disorders [9]. ES also shows efficacy in promoting regeneration in muscle and bone [ 10, 11] . *In vitro*, ES also influences several types of cell behavior, including proliferation, differentiation, and migration [ 12, 13] . Hence, the activation of neurons through ES is a promising approach.

The success of SCS and DBS in the treatment of dysfunctional diseases has led to the exploration of their underlying mechanisms. Neurons receive and spread electrical signals at a rapid conduction rate during internal communication, and this communication is characterized by an all-or-none property. The nerve impulses can be modeled as an electrical circuit via the Hodgkin-Huxley (HH) model, and the cell membrane and the attendant ion channels are considered capacitors and resistors, respectively [14]. Therefore, the application of the different modes of ES to influence neuronal behavior and determine the optimal parameters for stimulation could provide a better understanding of the molecular mechanisms.

Previous studies revealed morphological differences in cultured cortical neurons between the control and experimental groups, which shows a potential reason for axon branching by increasing the secretion of neurotrophins. Modifications to the synaptic structure and function are components of synaptic plasticity, which is essential to memory and learning [15]. Brain-derived neurotrophic factor (BDNF) and other neurotrophins are thought to contribute to plasticity by regulating synaptic strength [ 16, 17] . Furthermore, a considerable body of evidence supports the role of BDNF in the regeneration, differentiation, and survival of neurons [18].

In this study, we investigated the effects of microcurrent electrical stimulation (direct/alternating current stimulation, DCS/ACS) on the behavior of primary cortical neurons. We developed a conducting surface based on indium tin oxide (ITO) glass that allows neuron outgrowth and neurite extension to assess whether the application of microcurrent stimulation in culture medium provides any benefits to neurons. Platinum electrodes, which are highly stable and resistant to oxidation, were connected to ITO conductive glass for the application of DCS and ACS, and the effects on primary neuron viability, growth, and expressions of synaptic formation markers under different types of stimulation settings were investigated. We did not find significantly different expression levels of BDNF, β-NGF, and NT-3 in the culture medium. We then performed mRNA sequencing and data analysis to identify the potential mechanisms of ACS/DCS on cortical neurons. Similar to the functional electrical stimulation (FES) scenario, ACS extensively changes neuronal transcription profiles. These changes at the molecular and genetic levels are particularly prominent in synapse plasticity, which is related to refined network neuronal circuit reform. Moreover, we found that these changes may be attributed to an increase in GAP43 expression through the PI3K/AKT pathway, which plays an important role in axon branching and sprouting. These findings advance our understanding of the mechanisms of action underlying the therapeutic effects of ES, such as DBS and SCS.

## Materials and Methods

### Electrode preparation and design

**Figure 1:**
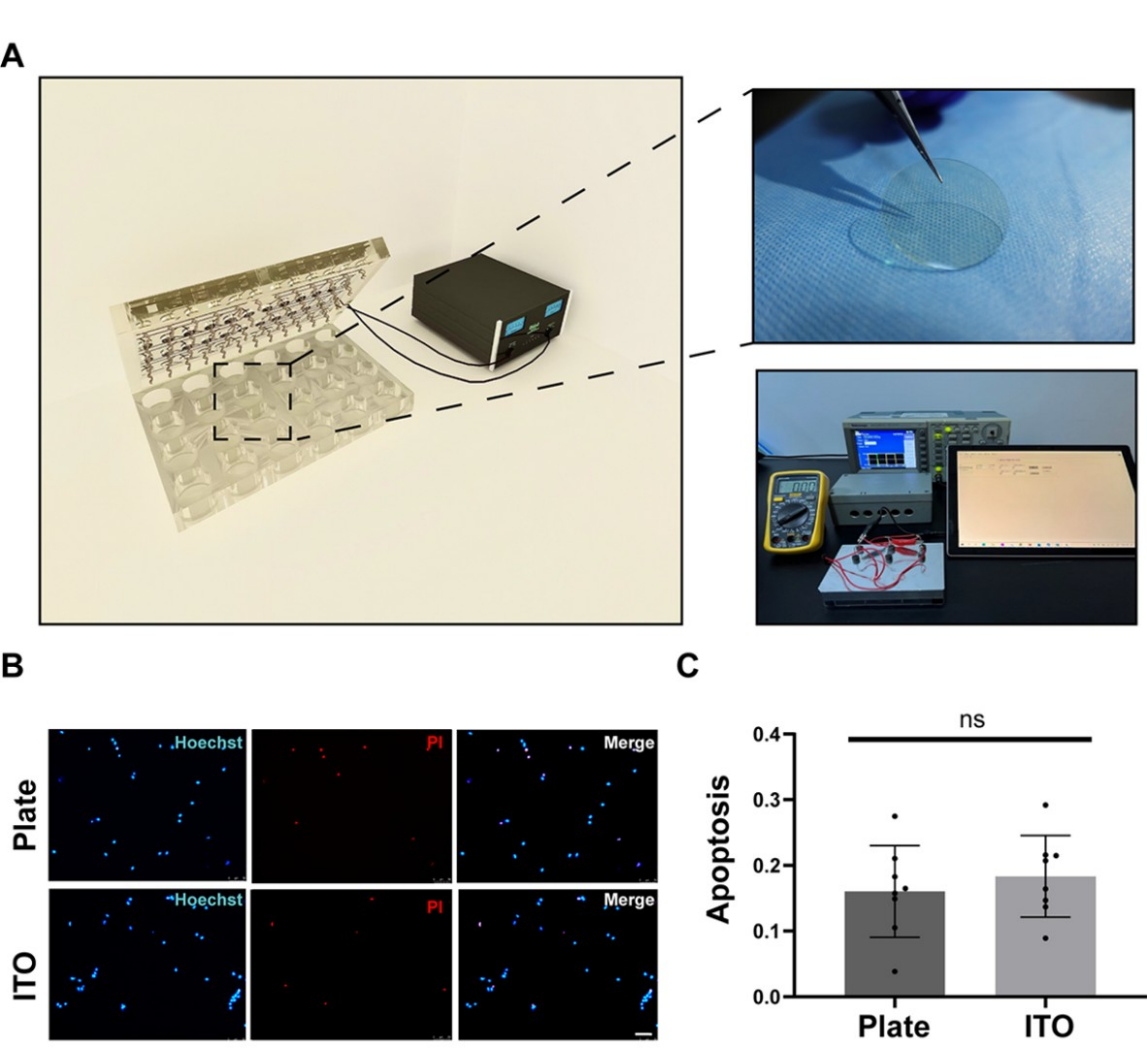
Figure 1 Construction of the cell electrical stimulation platform (A) Design of the ES system of the chamber with platinum spring electrodes integrated into the lid of a 24-well plate. (B,C) Cell death was quantified by Hoechst/PI staining ( n=8). Scale bar: 50 μm. Data are expressed as the mean±SD.

The bottom working ITO glass with a resistance of 20 Ω and a size of 9.6 cm ^2^ and 2 cm ^2^ was used as the electrically conductive surface. The conductivity of the ITO glass surface was measured prior to use. Furthermore, the size and shape of the ITO glass were designed according to the size of the cell culture plate, which makes its placement and sterilization easy. We then developed an aluminum chamber slide system. The Pt and reference electrodes were placed on the lid of the chamber, and the tip of the electrode was designed to have a spiral shape to guarantee stable contact with the ITO glass ( Figure 1A).

### DCS and ACS

DCS was delivered with a PC20 WEN 8-channel high-precision and programmable electrical stimulation device (Aixin, Shanghai, China). ACS was delivered with a Tektronix AFG3051C single-channel function generator. Direct and alternating currents were then applied to the working ITO/glass electrode ( Figure 1A). To measure the current, a digital multimeter (Fluke, Shanghai, China) was connected to the circuit, which comprised the stimulating ITO electrode and stimulator connected in series.

### Cortical neuron cultures and purity

Cultures of primary cortical neurons were generated from embryonic day 16‒18 Wistar rat embryos as described previously [19] and were plated (10 ^5^ neurons per 2-cm ^2^ well). The cultures were maintained in Neurobasal medium (Gibco, New York, USA) supplemented with B27-A (Gibco) and GlutaMAX (Gibco). The purity of the primary cortical neurons was determined by staining with anti-beta III tubulin antibody (Abcam, Cambridge, UK) and goat anti-rabbit Cy3 (1:1000) antibody (Beyotime, Shanghai, China). The proportion of Beta III tubulin-positive cells to total cells was quantified. This study was approved by the Tianjin Medical University General Hospital Ethics Committee (IRM-DWLL-2021110).

### TUNEL assay

A one-step TUNEL assay kit and Hoechst/PI (Beyotime) were used according to the manufacturer’s protocol. Briefly, the neurons were fixed and then permeabilized. The TUNEL reaction mixture was added to neurons after wash with PBS and then incubated in the dark for 1 h at room temperature. Neurons were stained with Hoechst/PI for 15 mins in the dark. After staining, neurons in the culture plates were analyzed under a fluorescence microscope.

### Morphological analysis

The branches and network complexity were measured by examining the neurite number and length. As described previously [ 20, 21] , morphological analyses, including Sholl analysis, were performed. The dendrites and axons (neuronal processes) of cultured neurons were counted manually. In each experiment, five microphotographs were randomly obtained per well and were subjected to analysis using ImageJ software (NIH, Bethesda, USA). The Simple Neurite Tracer plugin was used for the semiautomatic tracing of neurons.

### Measurements of cortical neuron electrical activity

MEA plates (Axion Biosystems, Atlanta, USA) were sterilized with alcohol, coated with laminin and washed with PBS before seeding. Cortical neurons were isolated and plated at the center of the MEA plate (approximately 10 ^5^ cells per well). The neurons were incubated for 14 days before being used for measurements. The MEA plates were inserted into the Maestro Edge (Axion Biosystems) to monitor and record the baseline electrical activity of neurons for 5 min. Further analysis was performed using AXiS Navigator and Neural Metric Tool software (Axion Biosystems).

### Immunofluorescence assay

The neurons were fixed with 4% paraformaldehyde (Beyotime) for 30 min, permeabilized and blocked for 1 h. The neurons were washed with PBST and then incubated overnight with anti-beta III tubulin antibody (rabbit anti-beta III tubulin, 1:1000; Abcam). Finally, fluorescence images were obtained under a Leica inverted microscope (DMi8; Leica, Solms, Germany).

### Western blot analysis

Neurons were collected and then lysed using RIPA lysis buffer (Beyotime) supplemented with a phosphatase inhibitor cocktail (Roche, Basel, Switzerland) on ice. After measurement of the protein concentration, 12.5% SDS–PAGE gels (EpiZyme Biotechnology, Shanghai, China) were used to separate equal amounts of protein from each group, and the proteins were transferred to PVDF membranes with a thickness of 0.2 μm. The membranes were blocked for 1 h with 20% skimmed milk powder (Difco ^TM^; BD, San Diego, USA) or 5% BSA (Solarbio, Beijing, China) in TBST and then incubated overnight at 4°C with the following primary antibodies: rabbit anti-GAPDH (1:1000; Abcam), rabbit anti-GAP43 (1:1000; Abcam), rabbit anti-PI3 kinase p110α (C73F8) (1:1000; CST, Danvers, USA) and rabbit anti-phospho-PI3 kinase p85 (Tyr458)/p55 (Tyr199) (1:1000; CST). After several washes, membranes were incubated with the appropriate HRP-labelled goat anti-rabbit IgG (H+L) secondary antibodies (1:1000; Beyotime) at room temperature for 1 h. Finally, enhanced chemiluminescence (Immobilon; Millipore, Billerica, USA) detection was performed. Western blotting signals were acquired using an image analysis system (SYNGENE, Cambridge, UK) and quantified using ImageJ software.

### RT-qPCR

The isolation of total RNA was conducted using TRIzol reagent (Invitrogen, Carlsbad, USA). mRNA was reverse transcribed using oligo dT primers and a FastKing One-step RT-qPCR kit (Tiangen, Beijing, China). A total of 2 ng of RNA was collected from each sample for reverse transcription and cDNA synthesis. One microliter of each cDNA sample was used for PCR amplification. RT-PCR was performed on a LightCycler (Roche) using PCR primers for *Nr4a1*, *Egr*, *Fos*, *NT-3*, *GAP43*, *β-NGF*, *BDNF*, *Ager*, *Dmpk*, and *Arc*, which were synthesized by Sango Biotech (Shanghai. China). The PCR conditions were as follows: 94°C for 2 min, 30 cycles of amplification consisting of 94°C for 45 s, 55°C for 45 s and 72°C for 1 min, and 72°C for 5 min. The mRNA levels were normalized using *GAPDH* as the internal control. Sequences of primers are presented in Supplementary Table S1.

### Extraction and library construction

The purity of RNA from each sample was confirmed by the 260/280 ratio using a NanoDrop ND-1000 (NanoDrop, Wilmington, USA). The RNA integrity was assessed using the Bioanalyzer 2100 (Agilent, Santa Clara, USA) with an RIN value >7.0 as the threshold. Poly(A) RNA was purified from 1 μg of total RNA using Dynabeads Oligo(dT)25-61005 (Thermo Fisher, Waltham, USA) with two rounds of purification. In the magnesium RNA Fragmentation Module, the poly(A) RNA was fragmented into small pieces for 5‒7 min at 94°C. The cleaved RNA fragments were then reverse-transcribed to generate cDNA. An A-base was then added to the blunt ends of each strand to prepare the products for ligation to the indexed adapters. T-base overhangs were present on each adapter to bind DNA fragments with A-tails. Single-index or dual-index adapters were ligated to the fragments, and size selection was performed with AMPure XP beads (Beckman Coulter, Pasadena, USA). After the heat-labile UDG enzyme treatment of the U-labeled second-stranded DNAs, the ligated products were amplified by PCR with the following conditions: initial denaturation for 3 min at 95°C, 8 cycles of denaturation at 98°C for 15 sec, annealing at 60°C for 15 sec, and extension at 72°C for 30 sec and a final extension at 72°C for 5 min. The average insert size for the final cDNA library was 300±50 bp. Finally, 2×150-bp paired-end sequencing (PE150) was performed on an Illumina NovaSeq™ 6000 (Illumina, San Diego, USA) following the manufacturer’s protocol.

### Bioinformatics analysis of RNA-seq

Fastp software was used to remove reads containing adaptor contamination, low-quality bases and undetermined bases with default parameters. Afterward, the sequence quality was also verified using fastp. HISAT2 ( https://ccb.jhu.edu/software/hisat2) was used to map reads to the reference genome of Rattus norvegicus GRCh38. The mapped reads of each sample were assembled using StringTie ( https://ccb.jhu.edu/software/stringtie) with the default parameters. After all transcriptomes from all samples were merged, gffcompare ( https://github.com/gpertea/gffcompare/) was used to reconstruct a comprehensive transcriptome. After the final transcriptome was generated, StringTie was used to estimate the expression levels of all the transcripts. With StringTie, FPKM (FPKM=[total_exon_fragments/mapped_reads (millions)× exon_length (kB)]) was used to calculate the expression levels of mRNAs. The mRNAs with a fold change >2 or <0.5 and a *P* value<0.05 from a parametric F test comparing nested linear models were selected as differentially expressed mRNAs using the R package edgeR ( https://bioconductor.org/packages/release/bioc/html/edgeR.html). The sequencing data have been uploaded onto the Gene Expression Omnibus with the accession no. GSE207117.

### Statistical analysis

The data were analyzed using SPSS (version 23.0) and GraphPad Prism (version 8.0.2). Fiji was used to quantitatively analyze the images. Sample size calculation was performed to determine the appropriate sample size ( http://powerandsamplesize.com/). The group sizes were calculated to provide at least 80% power. Each dot represents the number (N) of samples per group. The data normality was evaluated with the Kolmogorov–Smirnov test, and the variance homogeneity was evaluated using Levene’s test. To compare two groups of data, unpaired *t* tests were used. If the data were normally distributed and the variance was homogeneous, one-way ANOVA followed by the post hoc Bonferroni′s test was performed. If the data were not normally distributed or variance homogeneity was not met, the Kruskal–Wallis test was performed followed by the post hoc Mann–Whitney U test. The nonparametric data are displayed as the medians and interquartile ranges, and the other data are displayed as the means±standard deviations (SDs). *P*<0.05 was considered to indicate a significant difference. All the samples were assayed in a randomized manner. All analyses were conducted blinded to the experimental or control group.

## Results

### Establishment of the electrical stimulation system and biological safety evaluation of the ITO glass electrode

The stimulation current was generated using an alternative or direct current stimulator. The pattern of waveforms was set in the software kit or the signal generator. To measure the current flowing through the ITO electrode surface, a digital multimeter was then connected in series to monitor the current fluctuation during the stimulation.

The electrical stimulation system design is shown in Figure 1A. We used ITO-coated glass to decrease the culture medium resistance. All neurons were grown on glass, and all electrodes were connected in series to ensure that the strengths of the stimulus currents in every well were the same. Before each stimulation, the device was tested in the conducting state. We performed Hoechst/PI staining to test whether the exposure of neurons to ITO glass would exert cytotoxic effects on the neurons. Cell death was quantified by direct counting of the Hoechst-stained nuclei after two weeks of culture (DIV 14). Hoechst/PI staining revealed that ITO glass did not induce any increase in the cell death rate ( Figure 1B,C). The results confirmed that the ITO glass culture system is not toxic to neurons.

### Investigation of optimal stimulation parameters

**Figure 2:**
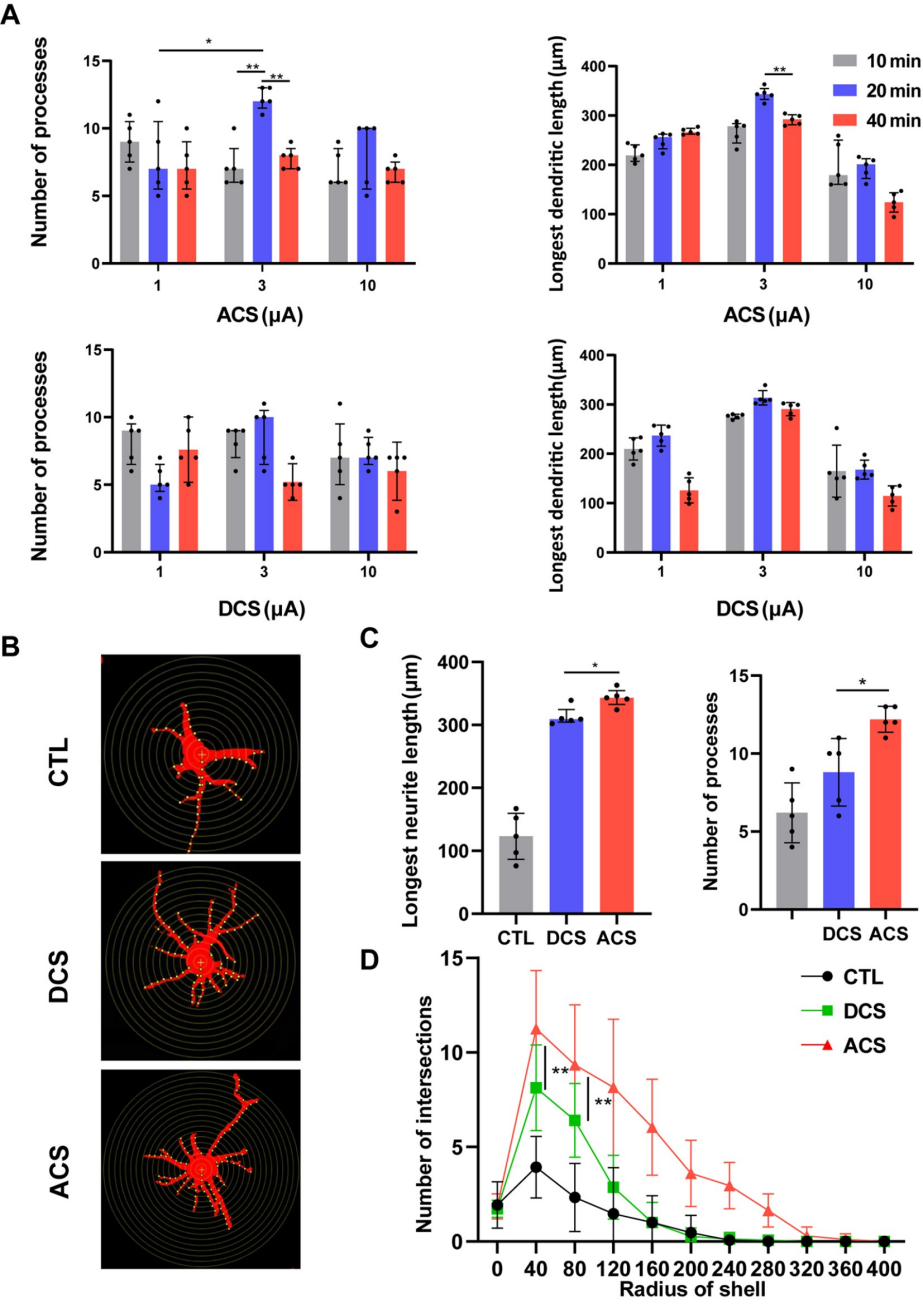
Figure 2 Electrical stimulation (ES) promotes axonal outgrowth of neurons (A) Quantitative measurement of the lengths and numbers of branches of outgrowing neurites obtained with different parameters. (B) Eight-bit tracing with digitally applied concentric rings spaced at distances of 20 μm centered around the soma center. (C) ES accelerates the outgrowth of axons. (D) Sholl profiles of the CTL, DCS, and ACS groups. n=5. * P<0.05, ** P<0.01.

In a previous study, preosteoblasts subjected to 3-μA direct current stimulation for two weeks showed increased cell viability and enhanced cell proliferation and metabolism [22]. Tanamoto *et al*. [23] found that an ITO electrode-based *in vitro* stimulation device evoked a Ca ^2+^ channel response in PC12 cells depending on the current flowing with a threshold of 60 μA. Based on previous results, we chose to analyze the optimized stimulation parameters based on the settings of 1, 3, and 10 μA and ACS or DCS for 10, 20, and 40 min per day for 7 days. For ACS, the frequency was set to 20 Hz, which is the widely recognized paradigm for chronic FES [24]. With a width of 25 ms, the above settings produced a square wave voltage of 4‒6 Vpp. Quantitative measurement of the lengths and numbers of branches of outgrowing neurites was performed under different ES parameters. The neurites were longer in almost all experimental conditions, and the length was correlated with the intensity and time of the stimulation. The exposure of cultures of neurons to alternating current electrical stimulation at 3 μA for 20 min greatly enhanced the degree of neurite outgrowth ( Figure 2A). The optimized alternating and direct current intensity and duration were identified in the present study. Despite the growing body of evidence on electrical stimulation, a direct comparison between ACS and DCS is needed. Therefore, we directly compared the effects of ACSs and DCSs on neurons in culture. Sholl analysis is an extensively used quantitative method to analyze the complexity of dendritic arbors. The Sholl profiles of cell tracings are shown in Figure 2B. The neurons featured a more complex, branched, and elongated neurite after ACS than after DCS and ITO only. ACS (3 μA, 20 min) produced the longest neurites and the highest number of processes ( Figure 2C,D). Intersections were counted at 40-μm intervals from the soma center to a radius of 400 μm. The Sholl profiles showed that neurite arborization was higher in the ACS group than in the DCS and CTL groups. The total neurite length, number of branch points and maximum branch level of neurons after ACS treatment were significantly greater than those in the other groups.

### ACS elicits an increase in the spontaneous electrical activity of neurons

**Figure 3:**
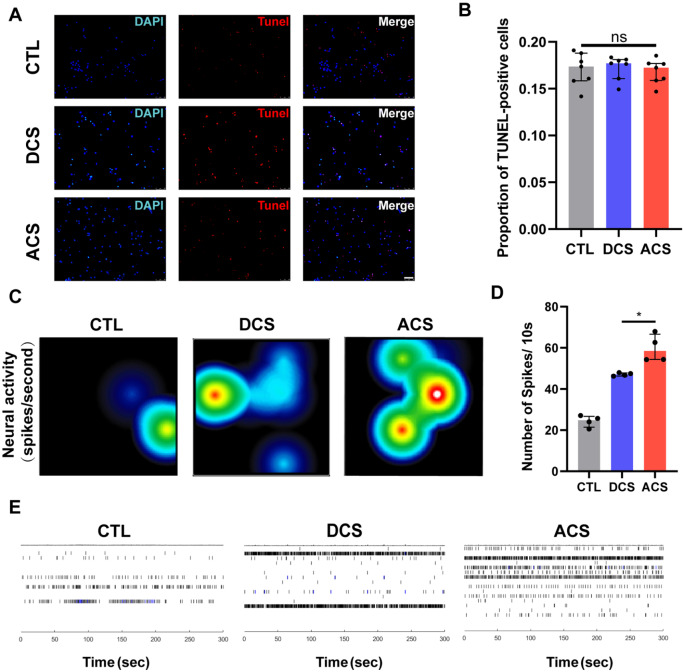
Figure 3 ACS elicits an increase in the spontaneous electrical activity of neurons (A) TUNEL staining of neurons under electrical stimulation. (B) Statistical analysis of dead neurons in the ischemic penumbra ( n=7). (C) Heatmap showing the evolution of the average activity. (D) Statistical analysis of the number of spikes ( n=4). (E) Raster plots of the electrophysiological response of the neurons. Scale bar: 50 μm. * P<0.05.

Cell death under different stimulation paradigms was examined by TUNEL staining. Exposure to DCS and ACS did not induce cell death in neurons ( Figure 3A,B). To evaluate the effects of ES, we further investigated the rate of change in network excitability and connectivity. MEA is a grid of microelectrodes that can measure neuronal activity without disrupting the cells. Neurons form spontaneous networks during development and communicate across synapses. When the above neuron fires an action potential, a spike signal simultaneously appears. Through the electrodes on the array, spikes are simultaneously detected to monitor the activity and network behavior. The MEA was used to stimulate the neurons and measure the electrical activity. Neurons were seeded in 24-well MEA plates, and recordings were acquired 14 days after seeding. ACS increased neuronal activity and significantly increased the number of spikes ( Figure 3C,D). A raster plot is a simple way to visualize the network behaviors of the neuron. We found that exposure to ACS elicited a significant increase in the spontaneous electrical activity of neurons ( Figure 3E).

### ACS increases GAP43 expression but does not change neurotrophin secretion

**Figure 4:**
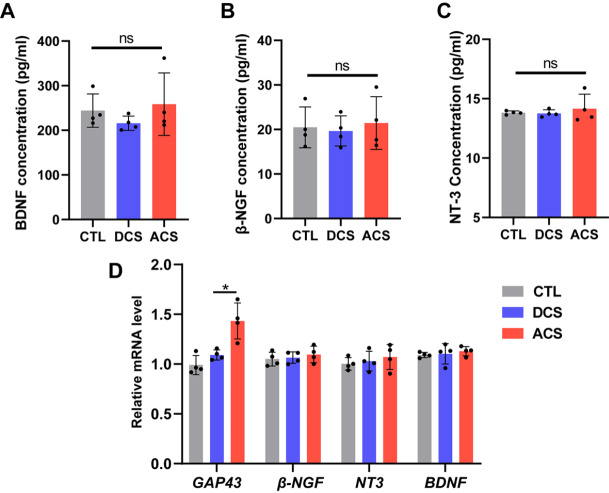
Figure 4 ACS increases GAP43 expression but does not change neurotrophin secretion (A‒C) Measurement of neurotrophin expression levels by ELISA. (D) Quantitative PCR quantification of the expression levels of GAP43, β-NGF, NT-3, and BDNF. n=4. * P<0.05.

Representative immunofluorescence photomicrographs showed significant increases in the numbers and lengths of processes, which may involve BDNF and other neurotrophins, according to the recent literature [ 25– 28] . In glutamatergic and GABAergic synapses, BDNF is crucial for growth, development, and plasticity. BDNF is also involved in serotonergic and dopaminergic neurotransmission by modulating neuronal differentiation and acts as an autocrine and paracrine factor on pre- and postsynaptic target sites, which is essential for transforming synaptic activity into long-term synaptic memories. Therefore, we speculated that the active metabolism and growth of neurons in the ACS group may be due to the activation of neurotrophin secretion. In addition, other neurotrophins, such as β-NGF and NT-3, act as paracrine and autocrine factors and may be detected in the supernatant of the culture plate. Therefore, the supernatants of the three groups were collected to measure the BDNF, β-NGF and NT-3 levels by ELISA. We found no difference in the concentrations of BDNF, β-NGF and NT-3 among the three groups ( Figure 4A‒C), and no difference in the corresponding expression levels was found ( Figure 4D). This finding indicates that neuronal outgrowth and neurite extension after ACS and DCS may involve other mechanisms, which suggests that additional work is needed to elucidate the mechanisms underlying these distinct changes and the functional consequences.

As a neuron-specific growth-associated protein, GAP43 is selectively distributed to the axonal domain in developing neurons. This protein has also been implicated in neuronal development, axonal regeneration, and synaptic plasticity. As shown in Figure 4D, RT-PCR analysis revealed that *GAP43* gene expression was significantly increased in the ACS group. The above results indicate that ACS could promote the expression of *GAP43* mRNA. However, the mechanisms that regulate axonal outgrowth of neurons remain unclear and require further investigation.

### Identification of DEGs in the DCS and ACS groups

**Figure 5:**
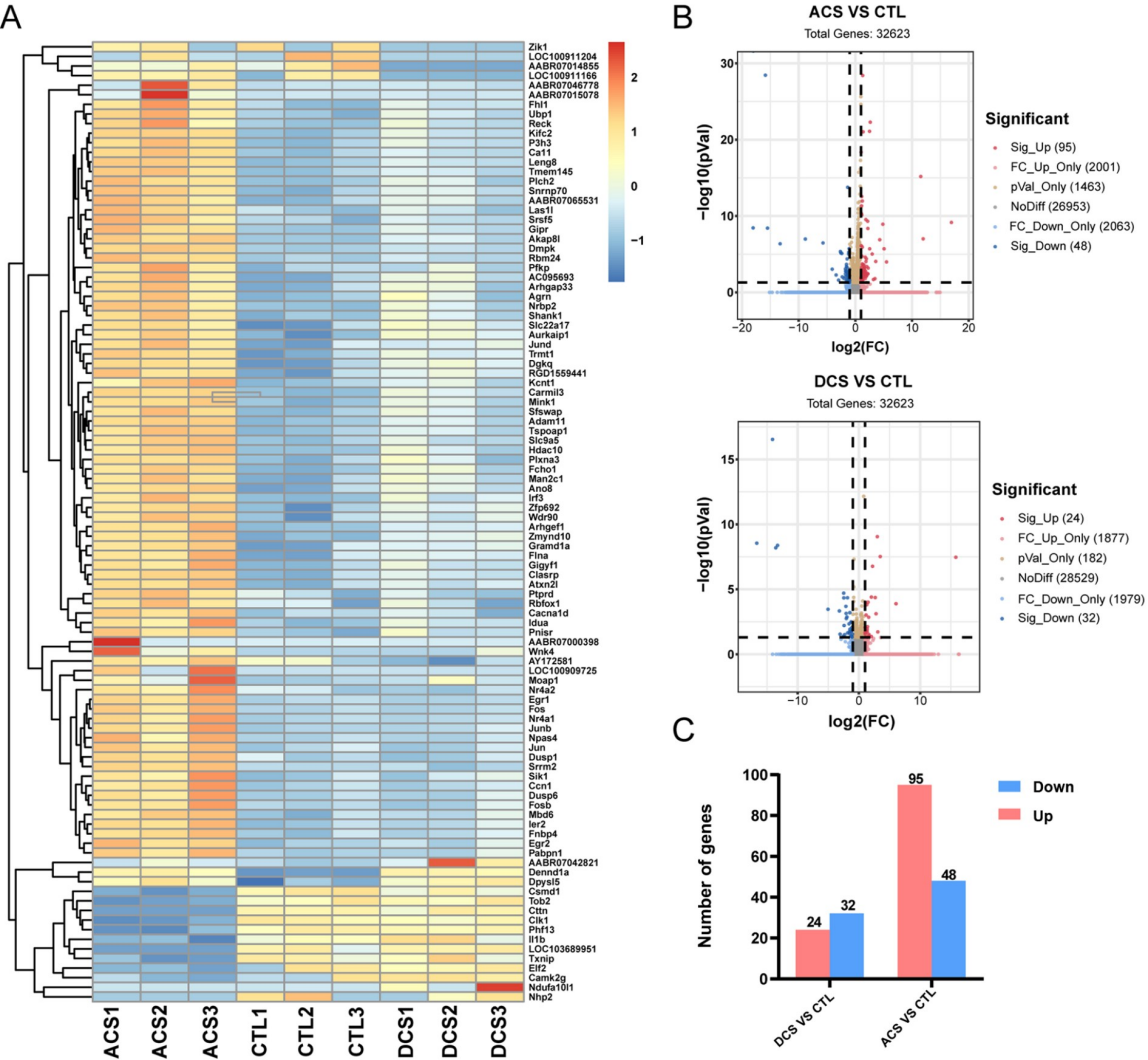
Figure 5 Identification of DEGs in the DCS and ACS groups (A) Heatmap of the DEGs. (B) Volcano plot of the identified DEGs. (C) Differentially expressed genes in the different groups.

To better understand how ES affects neurons, cortical neurons from the control, DCS, and ACS groups were further collected for RNA sequencing and bioinformatics data analysis to identify associated genes. In the heatmap, the top 100 DEGs were selected, as shown in Figure 5A. The volcano plot, which was used to visually represent the identified DEGs, is presented in Figure 5B. According to the thresholds of *P*<0.05 and |logFC|>1, a total of 143 DEGs that exhibited different levels between the ACS and control groups were selected, and these included 95 upregulated DEGs and 48 downregulated DEGs. A total of 56 DEGs that differed in expression between the DCS and control groups, including 24 upregulated DEGs and 32 downregulated DEGs, were selected. The cluster analysis of the top DEGs shown in Figure 5C revealed that these DEGs exhibit significant clustering between the ACS and DCS group cell samples.

### Activation of neural regeneration genes after ACS

**Figure 6:**
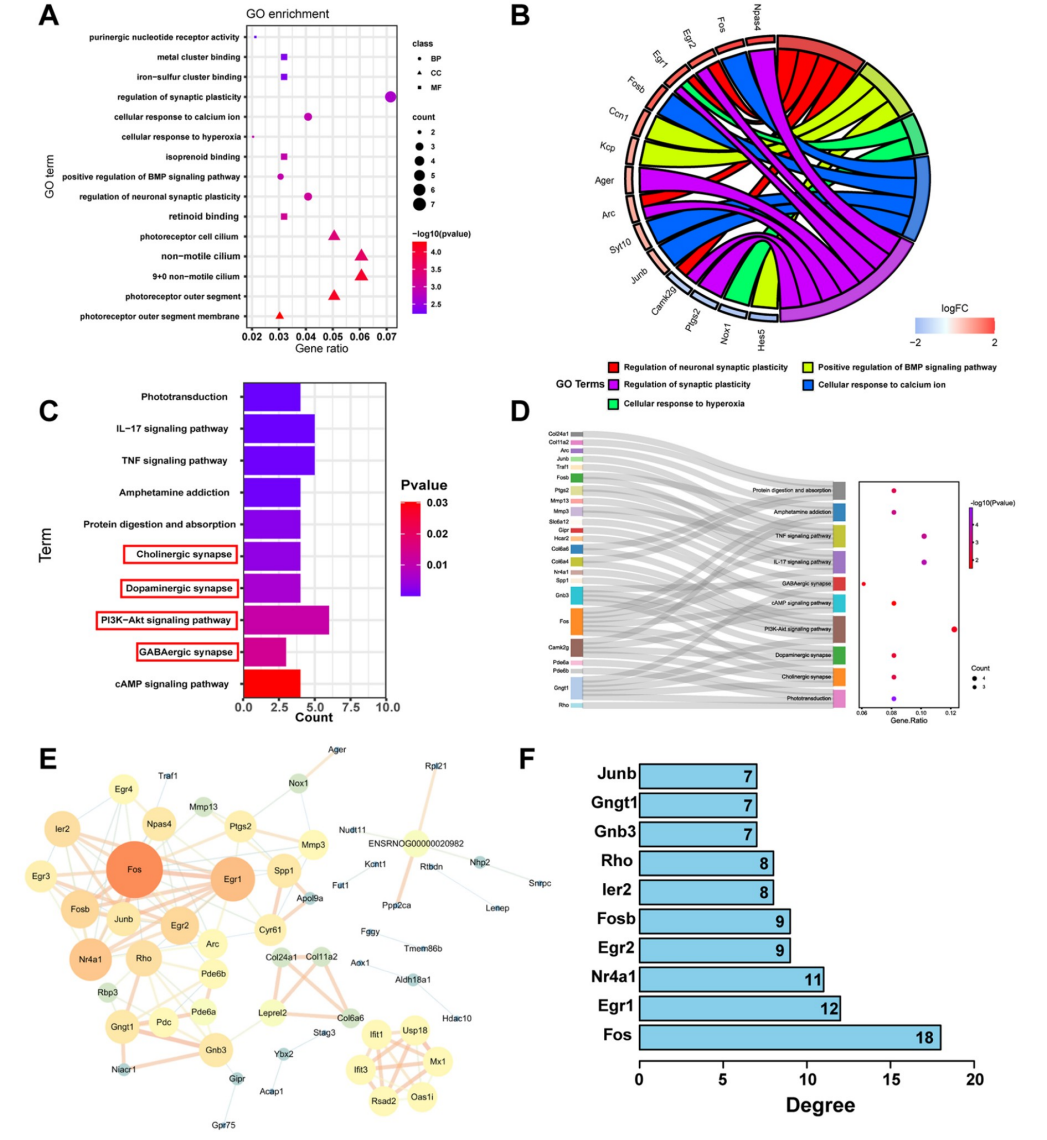
Figure 6 Activation of neural regeneration genes after ACS (A,B) Results from the GO analysis with respect to biological processes. (C) KEGG analysis. (D) The target gene was mapped to the pathway to obtain a Sankey diagram. The dot plot shows the total number of genes in each enriched pathway. (E) Structure of the differentially expressed gene protein interaction network. (F) The top 10 most strongly connected PPI nodes were identified as hub proteins.

A GO functional enrichment analysis showed that DEGs were enriched in biological processes related to synaptic plasticity and cellular response to calcium ions, in the molecular function terms metal cluster binding, iron-sulfur cluster binding, isoprenoid binding, and retinoid binding, and in the cellular component terms photoreceptor cell cilium, nonmotile cilium, and photoreceptor outer segment ( Figure 6A,B). KEGG pathway enrichment analysis and Sankey diagram revealed that these DEGs were mainly enriched in various pathways, including the PI3K-Akt signaling pathway, IL-17 signaling pathway, phototransduction, cholinergic synapse, dopaminergic synapse, GABAergic synapse, and cAMP signaling pathway ( Figure 6C,D). The GO and KEGG analyses revealed that the genes in the module were related to the regulation of neuronal synaptic plasticity, cellular response to calcium ions and the PI3k-Akt signaling pathway.

Subsequently, to determine the major proteins, PPI networks were established using STRING data of the differentially accumulated proteins identified from the comparison of the ACS group with the control group. A total of 58 differentially accumulated proteins were filtered in this analysis ( Figure 6E). The PPI network consisted of 58 nodes and 121 edges. The following hub proteins were selected from the top 10 most strongly connected PPI nodes: Fos, Egr1, Nr4a1, Egr2, Fosb, ler2, Rho, Gnb3, Gngt1 and Junb ( Figure 6F). Among these 10 proteins, Fos exhibited the highest connectivity in the PPI network.

### ACS upregulates related gene transcription and is involved in PI3K/AKT/GAP43 upregulation

**Figure 7:**
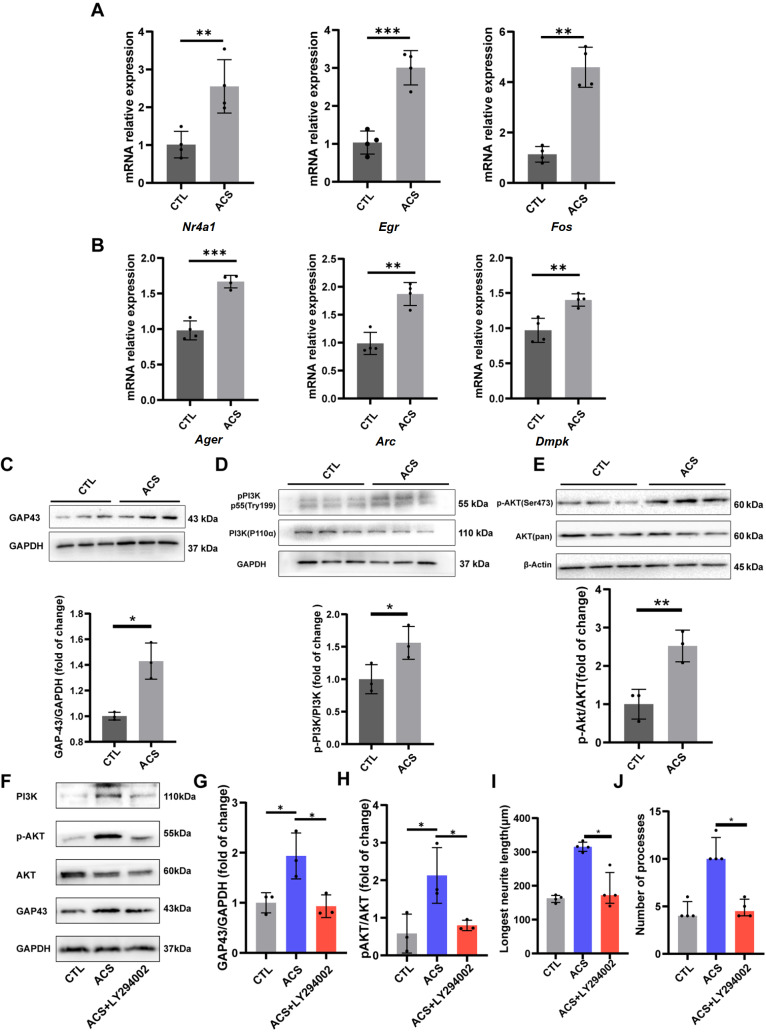
Figure 7 The effects of ACS on neurite growth might be associated with the PI3K/Akt/GAP43 signaling pathway (A) Quantitative PCR quantification of the expression levels of Egr, Fos, and Nr4a1 ( n=4). (B) Quantitative PCR quantification of the expression levels of Ager, Arc, and Dmpk ( n=4). (C‒E) Western blot analysis was performed to assess the activation of the GAP43/PI3K/AKT signaling pathway by detecting the expressions of GAP43, PI3K, p-PI3K, AKT and p-AKT ( n=3). (F‒H) Western blot results of the PI3K/AKT/GAP43 pathway and blockade by the PI3K-AKT pathway inhibitor LY294002. (I,J) LY294002 attenuated the outgrowth of neurons ( n=4). * P<0.05, ** P<0.01, *** P<0.001.

Based on our sequencing analysis, we performed qRT-PCR to validate our results. As predicted by the hub proteins, ACS significantly increased the transcription of the *Nr4a1*, *Erg1* and *Fos* genes ( Figure 7A). Advanced glycosylation end product-specific receptor (Ager) is involved in the transmission of synaptophysin [29]. *Arc* encodes cytoskeletal-associated proteins that are expressed in neurons. Dmpk is the DM1 protein kinase responsible for regulating synapse structure formation and cognitive function. ACS led to significantly higher levels of *Ager*, *Arc*, and *Dmpk* gene transcription than those found in the control group ( Figure 7B).

GAP43 expression increases significantly during the development of neuronal processes involved in the outgrowth of axons [ 30, 31] . GAP43 is also the main component of motile ‘growth cones’, which play an important role in the generation of axonal and dendritic filopodia and form the tips of elongating axons [32]. The abovementioned information shows that ACSs can promote the expression of GAP43 at the transcriptional level. According to our KEGG pathway enrichment analysis of the DEGs, the PI3K-AKT pathway may be activated in this process, and previous studies by Ogai *et al*. [33] and Songtao *et al*. [34] also suggested that the PI3K-AKT signaling pathway may be involved in axonal regeneration by increasing GAP43 expression.

The protein expression of GAP43 was also increased in the ACS group, as determined by western blot analysis ( Figure 7C). To further investigate the factors that impact the morphological and gene transcription changes in neurons, the expression levels of p-PI3K, PI3K, p-AKT and AKT were measured by western blot analysis. p-PI3K (p55 Tyr199) expression was increased in the ACS group ( *P*<0.05) but not in the control group ( Figure 7C). We obtained the same results from our analysis of p-AKT expression; specifically, p-AKT (Ser473) expression was increased in the ACS group ( *P*<0.01 *vs*. the control) ( Figure 7E). Therefore, we speculated that GAP43 may regulate neuronal outgrowth by activating the PI3K/Akt signaling pathway. To verify this mechanism, the PI3K-Akt pathway-specific inhibitor LY294002 was used. The expressions of GAP43, PI3K, p-AKT and AKT decreased in the group treated with the PI3K inhibitor LY294002 ( Figure 7F‒H). Moreover, the effect of ACS on promoting the growth of neuronal neurites was attenuated by LY294002 ( Figure 7I,J).

## Discussion

In the present study, we directly compared the effects of ACS and DCS on primary cortical neurons. Given the intrinsic electrical characteristics of neurons, we selected primary cortical neurons. Both ACS and DCS enhance the dendritic complexity of cortical neurons, and ACS exhibited a better effect than DCS. Additionally, we investigated the formation of the neuron network after ES by evaluating the firing spikes depicted in the raster plots, which reflect the activity, synchrony, and oscillation of the neuron network. We confirmed that the neurite growth effects of ACS might be associated with the PI3K/Akt/GAP43 signaling pathway.

Neurotrauma, neurodegenerative diseases, and other central nervous system-related diseases threaten the lives of millions of people worldwide. ES plays an important role in neural regeneration and is widely used for the treatment of nervous system disorders caused by SCI [35]. ES could accelerate nerve repair and functional recovery in animal models and patients. Shapira *et al*. [36] found faster behavioral and electrophysiological recovery in response to brief functional electrical stimulation therapy (3 V, 20 Hz) after severe in-continuity experimental nerve injury. In the central nervous system, the use of epidural electrical stimulation (EES) as a neuromodulation technique has long been known to treat chronic pain after SCI. Song *et al*. [37] implanted electrodes into rats after complete SCI with stimulation parameters of 40 Hz, 100–900 μA, and biphasic rectangular pulses and observed effective restoration of locomotion function in rats.

At present, we recognize two primary forms of ES: ACS and DCS. Numerous preclinical studies at the cell molecular level have been conducted to probe the possible mechanisms of action. Previous studies have reported that PC12 cell lines exposed to ACS exhibit enhanced neurite elongation [38]. In addition, Farahani *et al*. [39] found that DCS enhances synaptic plasticity in CA1 pyramidal neurons by increasing postsynaptic somatic spiking.

However, despite the growing evidence showing the effectiveness of ES, the differences between the effects of ACS and DCS on cells are unclear. Most types of ES used in published studies were ACS. The underlying mechanism of the cellular response to ACS and DCS remains unclear. ACS might prevent the buildup of charge in the cellular environment. Therefore, a direct comparison between ACS and DCS is needed.

Previous investigations of the mechanisms of ES have focused on voltage-gated Ca ^2+^ channels, transmembrane potentials, and ion flow [40]. However, the possibility that ES directly regulates gene expression cannot be ignored. Neurotrophins, including BDNF, β-NGF, and NT-3, support neuronal development, maintenance, and plasticity. The regulation of BDNF expression in neurons is considered to be linked to electrical activity. As observed in previous studies, the electrical stimulation (20 Hz, 3‒5 V) of cultured spinal cord neurons promotes BDNF expression [41]. Neurotrophins have thus been suggested to be involved in an important mechanism through which ES promotes the neurite growth of neurons. However, in the present study, we did not observe any increased secretion of neurotrophins, such as BDNF, β-NGF, and NT-3, after ES. ES may act via other nonspecific mechanisms, and this possibility warrants further investigation.

The membrane-associated protein GAP43, a marker of axonal and synaptic growth, is involved in axonal elongation, regeneration, and neurotransmitter release [ 30, 42] . We found that ACS increased GAP43 expression in cultured cortical neurons. We sequenced the transcriptome to investigate the transcriptomic changes observed after ACS. The results of the KEGG pathway analysis showed that the PI3K/AKT pathway was significantly enriched after ACS, and the expression levels of p-PI3K and p-AKT were also upregulated after ACS. PI3K/AKT are reportedly involved in the ES-mediated regulation of cell growth and other specific cellular processes [43]. The results from the present study demonstrate that the effect of ACSs in neurons might be due to increased GAP43 expression through the PI3K/AKT signaling pathway. The impact of ACS could be blocked by the presence of the PI3K-Akt pathway-specific inhibitor LY294002. These results confirm that the effects of ACS on neurite growth are associated with the PI3K/Akt signaling pathway.

In summary, we explored the influence of ACS and DCS on the electrophysiological characteristics of cultured primary cortical neurons. We established a simple, safe, and effective electrostimulation culture platform that is amenable to further research and translation. It is particularly important to study the mechanism through which ES affects cells and to formulate effective clinical therapy parameters. Moreover, neuronal electrophysiological properties and axonal morphology after ACS and DCS were compared. Transcriptome sequencing showed changes in the neuronal cytoskeleton and associated pathways, such as axonogenesis, the shape of axons, the growth of neurites, and the branching of axons after ACS. Based on these findings, we further determined the association of ACS with the PI3K/AKT/GAP43 pathway.

## Supporting information

Supplementary

## Supplementary Data

Supplementary data is available at *Acta Biochimica et Biophysica Sinica* online.
