# Supplementary material for: Alternating current stimulation promotes neurite outgrowth and plasticity in neurons through activation of the PI3K/AKT signaling pathway: Neurite growth enhanced by alternating current stimulation via PI3K/AKT pathway
Source: Acta Biochim Biophys Sin (Shanghai). 2023 Oct 9;55(11):1718–29. doi: 10.3724/abbs.2023238 (PMC10679878; doi:10.3724/abbs.2023238)
Supplement: Supplementary [file Supplementary.pdf]

Supplementary Table S1. Sequences of primers used in qPCR

Gene	Forward primer (5′→3′)	Reverse primer (5′→3′)
GAPDH	AACGGCACAGTCAAGGCTGA	ACGCCAGTAGACTCCACGACAT
GAP43	AACGGCACAGTCAAGGCTGA	ACGCCAGTAGACTCCACGACAT
BDNF	CTTGGAGAAGGAAACCGCCT	GTCCACACAAAGCTCTCGGA
NT-3	AGCTTGTCCCAGAATCCAGC	GACAAGCATCAGTCCCACGA
β-NGF	GTCTGGGCCCAATAAAGGCT	CTGTGTACGGTTCTGCCTGT
Egr	CCTGAACTGGACCACCTCTACTCTC	TGGCGGCGATAAGAATGCTGAAG
Fos	CCTTCACCCTGCCTCTTCTCAATG	AGCCTTCAGCTCCATGTTGCTAATG
Ager	CTGCCTCTGAACTCACAGCCAATG	TCCTGGTCTCCTCCTTCACAACTG
Arc	TGTTGACCGAAGTGTCCAAGCAG	CATAGCCGTCCAAGTTGTTCTCCAG
Dmpk	AGACACCCTTCTACGCCGACTC	GACACAGCAGCCCACGAATGAG
Npas4	GCAGTCATGTACCGATCCACCAAG	GGCAGCAGTTCCTTGAGGTTCC
